# Long COVID and health-related quality of life: a systematic review of immune, inflammatory, and metabolic markers

**DOI:** 10.3389/fpubh.2026.1846407

**Published:** 2026-06-17

**Authors:** Dinara Turebekova, Bakhyt Kosherova, Zhaniya Dauletkaliyeva, Yelena Shayakhmetova, Alexandr Marchenko, Dmitriy Solyanov, Vaiva Hendrixson, Irina Kadyrova

**Affiliations:** 1 The Scientific Research Laboratory, Karaganda Medical University, Karaganda, Kazakhstan; 2 Karaganda Medical University, Karaganda, Kazakhstan; 3 School of Public Health, Karaganda Medical University, Karaganda, Kazakhstan; 4 Department of Neurology, Psychiatry and Rehabilitation, Karaganda Medical University, Karaganda, Kazakhstan; 5 Faculty of Medicine, Institute of Biomedical Sciences, Vilnius University, Vilnius, Lithuania

**Keywords:** Long COVID, health-related quality of life, chronic inflammation, clinical outcomes, immune dysregulation, immune parameters, inflammatory markers, metabolic parameters

## Abstract

**Introduction:**

Long COVID is known to be associated with prolonged multiple organ symptoms and decreased health-related quality of life (HRQoL), but the pathogenesis and relationship between immune, inflammatory, and metabolic biomarkers and HRQoL remains poorly understood. This systematic review aims to synthesize the evidence on the health-related quality of life of patients with Long COVID and to summarize the reported associations between health-related outcomes and biomarkers.

**Methods:**

We conducted a systematic search in PubMed, Web of Science, and Scopus in search of studies that evaluated immune, inflammatory, or metabolic biomarkers and HRQoL in patients with Long COVID. This review included case–control and cohort studies with a control group. The Rayyan tool was used to select studies, and full-text articles were evaluated for compliance with the criteria. Information was obtained on biomarkers, analytical methods, tools for assessing the HRQoL, and the relationship between HRQoL and biomarkers. Due to the high heterogeneity of the methods, the results were summarized in a descriptive form.

**Results:**

Ten studies were included, involving 1,078 patients with Long COVID and 768 healthy controls. Most studies that used various methods to assess HRQoL consistently reported long-term deterioration after COVID, with the greatest deterioration observed in the areas of physical functioning, vitality, fatigue, daily activities, pain, discomfort, and respiratory distress. The most frequently measured markers were related to inflammation and endothelial/coagulation pathways, including CRP, hsCRP, IL-6, TNF-*α*, D-dimer, and VCAM-1. Autoimmune, metabolic, intestinal barrier, and omics-based markers were measured less frequently, but indicated phenotype-specific biological heterogeneity. Only a few studies have identified a direct link between biomarkers and HRQoL. Lower HRQoL indicators were associated with increased neurotoxicity, decreased calcium levels, systemic inflammation, endothelial dysfunction, and signals from individual autoantibodies.

**Conclusion:**

Our results indicate that Long COVID is consistently associated with a long-term decline in HRQoL, especially in the physical and functional areas. The available data indicate the presence of a strong signal regarding inflammatory processes and endothelial coagulation, while autoimmune, metabolic, and genetic data indicate phenotype-specific heterogeneity.

**Systematic review registration:**

Unique identifier: CRD420251239371, URL: https://www.crd.york.ac.uk/prospero/display_record.php?ID=CRD420251239371.

## 1 Introduction

The number of COVID-19 cases and deaths has decreased significantly this year compared to the early years of the pandemic. However, the health effects caused by SARS-CoV-2 infection, commonly known as Long COVID, are becoming an increasingly serious public health problem (1). The predominant symptoms include headache, chronic fatigue, “brain fog”, shortness of breath, sleep disorders, and chronic pain that can last for months and significantly interfere with daily activities (2). According to the Household Pulse survey, 80.1% of patients with Long COVID reported some degree of activity restriction at the time of the survey, while 24.3% reported significant activity restriction (3).

Thus, health-related quality of life (HRQoL) has become an important clinical indicator of public health in the long-term spread of COVID-19, because it reflects not only the severity of symptoms, but also the degree to which persistent symptoms interfere with physical, psychological, and social functioning (4). In the study by Rodrigues et al. (5), the HRQoL of Long COVID patients was evaluated using the SF-36 and PSQI instruments. A reduction in vitality and mental health was observed, with sleep disturbances, anxiety, and headaches having the greatest impact on worsening. Importantly, there was discordance between traditional clinical outcomes and self-reported symptoms among some patients, suggesting that conventional clinical indicators may not adequately reflect biological processes underlying symptom severity and impairment of HRQoL.

Despite its increasing frequency and socioeconomic relevance, the pathogenesis and origin of Long COVID symptoms remain unclear (6). The disease is characterized by remarkable clinical heterogeneity. Among the possible approaches to addressing this challenge, there are searches for immune, inflammatory, and metabolic biomarkers that may clarify the persistence of symptoms and allow differentiation of phenotypes. Biomarker-based stratification can facilitate planning of follow-up and tailored rehabilitation for the long term in the post-communicable disease population (7, 8).

The authors emphasize the persistent activation of innate and adaptive immune pathways with increased levels of circulating pro-inflammatory cytokines such as interleukin 6 (IL-6), interleukin 1 beta (IL-1β) and tumor necrosis factor alpha (TNF-α). Altered T cell responses and dysregulation of interferon are also noted (8). Other authors also report a relationship between markers of systemic inflammation, including C-reactive protein (CRP), ferritin, and D-dimer (9). These markers are associated with worsening symptoms and a more severe course of the disease in patients (10).

For example, in patients with Long COVID, the pathophysiology of one of the most common symptoms affecting the quality of life of patients, namely headache, has not been sufficiently studied. The main probable mechanisms associated with persistent and debilitating headaches in patients with Long COVID include direct penetration of the virus into the brain, cytokines, interleukin storm, persistent activation of the immune system and a pro-inflammatory state (11–14).

However, the available evidence is still incomplete. Existing studies significantly differ in study design, population characteristics, time since acute infection, case ascertainment, biofluids, analysis platforms such as ELISA, multiplex assays, liquid chromatography coupled to tandem mass spectrometry (LC–MS/MS), and nuclear magnetic resonance (NMR), as well as the instruments employed to evaluate HRQoL. This variability hinders the direct comparison of results from different studies and restricts the possibility of quantitative synthesis. Importantly, there is still limited direct evidence connecting health-related outcomes to biomarkers in Long COVID, and the evidence is heterogeneous. Most studies have evaluated these outcomes separately rather than within a combined analytical framework; therefore, it is necessary to conduct a systematic and structured review to determine which immune, inflammatory, and metabolic biomarkers are most consistently associated with symptom burden and health-related impairment in adults with Long COVID.

This systematic review aimed to synthesize evidence from studies evaluating both biomarkers and HRQoL in adults with Long COVID, and to examine the extent to which biomarker abnormalities were reported in parallel with, or in relation to, reduced HRQoL and symptom severity.

## 2 Materials and methods

This systematic review was conducted in accordance with the PRISMA 2020 statement (Preferred Reporting Items for Systematic Reviews and Meta-Analyses) (15).

### 2.1 Protocol and registration

The review protocol was developed *a priori* and registered in the International Prospective Register of Systematic Reviews (PROSPERO) in 2026 (registration number: CRD420251239371).

### 2.2 Search strategy

A literature search was conducted in January 2026 using PubMed, Web of Science, and Scopus. The search strategy combined terms related to Long COVID, fatigue, persistent symptoms, health-related quality of life, and immune, inflammatory, and metabolic markers. Keywords were linked using Boolean operators (Supplementary Table 1). The search was limited to original articles published in peer-reviewed journals. All studies included in this review were selected according to predefined eligibility criteria. Inclusion and exclusion criteria were specified *a priori* using the PECO framework (Supplementary Table 2).

### 2.3 Eligibility criteria (inclusion and exclusion)

This systematic review included original observational studies (cohort and case–control) involving adults (≥18 years old) with laboratory-confirmed or clinically diagnosed SARS-CoV-2 infection who subsequently developed Long COVID or post-acute effects of SARS-COV-2 infection (PASC), defined as persistent symptoms for at least 12 weeks after the onset of acute infection. To be acceptable, the studies had to include an appropriate comparison group consisting of either clinically healthy people who had no history of COVID-19 or participants who had fully recovered from acute SARS-CoV-2 infection without persistent symptoms within 12 weeks. The studies should have recorded immune, inflammatory, and/or metabolic parameters measured in serum or plasma during the post-acute period, as well as quality of life indicators assessed using validated questionnaires.

We excluded studies conducted exclusively on animal models or *in vitro*, publications without data on biomarkers that can be extracted, or without HRQoL assessments, as well as non-original articles, including reviews, case reports, editorials, and conference abstracts with no access to the full text.

### 2.4 Study selection

Rayyan, a web-based tool for systematic review screening, was used to facilitate the selection of eligible studies. Subsequently, full-text articles were independently assessed for eligibility by two reviewers (DT and YS). Any disagreements regarding study inclusion or exclusion were resolved through discussion and consensus between the reviewers.

### 2.5 Risk of bias assessment

Risk of bias assessment was performed only for studies included in the review after full-text screening. Appropriate appraisal tools were selected according to the study design. Observational studies, including cohort and case–control studies, were assessed using the JBI Critical Appraisal Checklists. Risk of bias was evaluated independently by two reviewers (Supplementary Table 3), and any disagreements were resolved through discussion until consensus was reached.

### 2.6 Data extraction

The following data were extracted from the studies included in the review: reference, country, study design, population (*N*), definition of the control group, definition of Long COVID, sample type, laboratory markers, analytical method, and methods used to assess quality of life. After comparison of the extracted information, any discrepancies were resolved by a third author (IK). In addition, the reference lists of all included articles were manually screened to identify any further relevant publications, which were subsequently subjected to the same screening process.

### 2.7 Ethical considerations

Ethical approval was not required, as this study was based exclusively on the analysis of previously published data.

## 3 Results

### 3.1 Literature search

The electronic database search identified 3,076 publications. After removal of 1,020 duplicates, 2,056 records remained for title and abstract screening. Following the initial screening, 1,346 publications were excluded for not meeting the inclusion criteria.

Full-text assessment was conducted for 710 articles. Of these, 700 publications were excluded because they did not meet the inclusion criteria, including due to the absence of relevant biomarker data, lack of quality-of-life assessment, inappropriate study design, or the absence of a control group.

**Figure 1:**
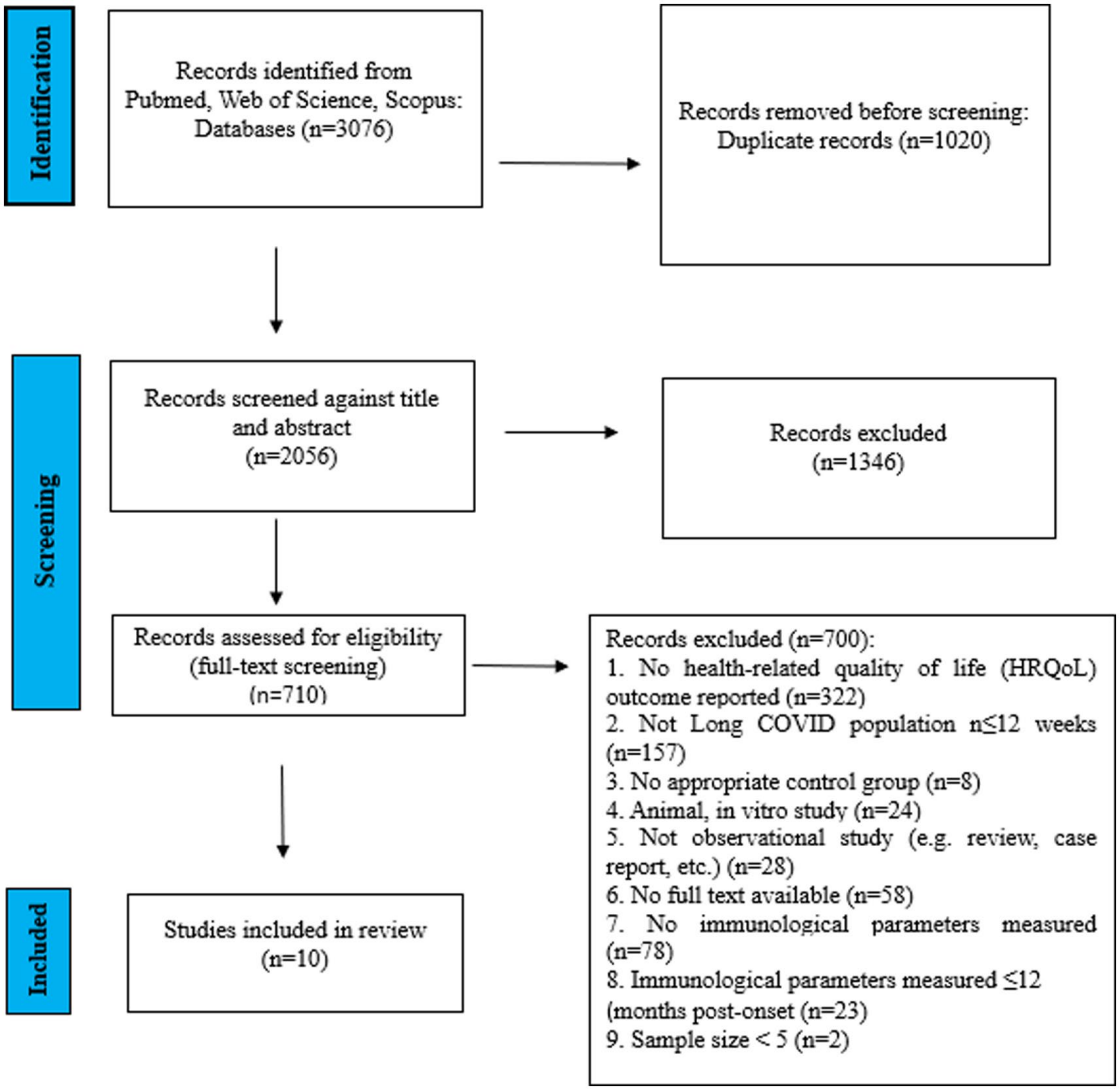
Flow diagram of search and selection of studies in the systematic review.

A total of 10 studies were ultimately included in the qualitative synthesis, the majority of which were observational in design. The study selection process is presented in the PRISMA flow diagram (Figure 1). The small number of eligible studies reflects strict inclusion criteria requiring both biomarker assessment and validated HRQoL outcomes within the post-acute phase.

### 3.2 Quality assessment

Assessment with the JBI Critical Appraisal Checklists showed that most included studies were of moderate methodological quality (80%), whereas 10% were rated as high quality and 10% as low quality. Confounding was identified in six studies, primarily because the comparison groups differed with respect to age, sex, body mass index (BMI), comorbidities, and severity of the acute infection, while adjustment for these factors was limited or insufficiently reported. In four studies, the control group was heterogeneous or comprised multiple control groups, and the source population differed across groups. Additionally, methodological concerns such as loss to follow-up and incomplete HRQoL data were identified in four studies.

### 3.3 Characteristics of included studies

The main characteristics of the studies are presented in Table 1. Countries included were Iraq, Thailand, the United States, Belgium, Canada, Malta, Spain, Germany, Austria, and China. Case–control studies were conducted in (16–20), while cohort studies were done in (21–25). The total sample size ranged from 88 to 358 participants, with varying proportions of women ranging from 27.9 to 79.5%.

**Table 1:** Study design and methodology of the included studies.

Reference	Country	Study design	Population (*N*)	Definition of control	Definition of long COVID	Public health outcomes	Sample type	Laboratory markers	Assay/measurement
Maes et al. (2022) (16)	Iraq	Case–control	Adults (≥18) *n* = 125	Healthy controls	WHO-based Long COVID, confirmed prior COVID; ≥2 symptoms affecting daily activities; symptoms lasting ≥3 months	WHO-QoL-BREF; HAMD; HAMA; BDI-II	Serum	IL-1β, IL-18, IL-10, caspase-1, MPO, AOPP, calcium, glucose, insulin	Spectrophotometric calcium; ELISA kits; glucose spectrophotometry; composite indices computed
Sangkaew et al. (2024) (25)	Thailand	Cohort	Adults ≥18 *n* = 300	Survivors without long COVID	Long COVID is defined by the WHO criteria (symptoms ≥3 months after infection)	EQ-5D-5L; Fatigue Score; Mini-Cog; GAD-7; PHQ	Blood	CBC; BUN, creatinine; electrolytes; CRP; D-dimer; LDH; IL-6; SARS-CoV-2 IgG (anti-NCP, anti-RBD)	Routine hospital laboratory testing; ELISA; CMIA (Abbott ARCHITECT)
Durieux et al. (2025) (17)	USA	Case–control	Adults ≥18 *n* = 179	SARS-CoV-2 nucleocapsid Ab negative	≥2 COVID-related symptoms starting >3 months	PROMIS-29; Global Health items	Blood; Plasma	Inflammation, endothelial: hsCRP, IL-6, sTNF-RI/II, sCD14, sCD163, ICAM, VCAM, D-dimer, oxLDL; gut integrity, translocation: I-FABP, zonulin, LBP, β-D-glucan	CLIA lab; ELISA
Polli et al. (2025) (22)	Belgium	Cohort	Adults ≥18 *n* = 358	Participants with no persistent symptom cluster (DSQ components)	Long COVID is defined as ≥1 symptom cluster (derived from DSQ items) with persistent symptoms, assessed 4–24 months after infection	SF-36; 6-min walk test, HADS; DSQ symptom clusters	Serum; Plasma	PBMC markers: LINE-1 global DNA methylation, telomere length, mtDNA copy number. Plasma cytokines (14-plex): Th1, Th2, Th9, Th17, Th22-related cytokines (e.g., IFN-*γ*, TNF-*α*, IL-2, IL-4, IL-10, IL-13, GM-CSF, IL-9, IL-17 family, IL-21, IL-22, MIP-3*α*). Serology: CRP, D-dimer, troponin T (hs), HbA1c, platelets, WBC	Pyrosequencing method; qPCR; Cobas 8,000 (Roche); MSD U-PLEX multiplex platform
Chiu et al. (2025) (23)	Canada	Cohort	Adults ≥18 *n* = 174	No Long COVID controls (prior confirmed SARS-CoV-2 infection but no Long COVID symptoms, recruited contemporaneously from same clinical network)	WHO Delphi Consensus criteria; persistent symptoms >12 weeks after SARS-CoV-2 infection	St George’s Respiratory Questionnaire, COPD Assessment Test, Fatigue Assessment Scale, mMRC dyspnea, Leicester Cough Questionnaire, Post-COVID-19 Functional Status.	Blood; Serum	CRP, IL-1*β*, IL-6, IL-8, TNF-α, D-dimer; endothelial dysfunction markers ICAM-1 and VCAM-1; hematology (WBC subtypes, NLR); autoantibodies, ENAs including anti-SS-B. Cytokines (IL-1β, IL-6, IL-8, TNF-α) and sputum ANAs, ENAs	Ella Automated Immunoassay System; IMTEC-ANA-LIA-MAXX
Xuereb et al. (2025) (18)	Malta	Case–control	Adults ≥18 *n* = 174	Negative COVID IgG at recruitment	Post-acute symptoms assessed at 6 months (NICE)	SF-36	Blood	hsCRP, vWF activity, troponin I, NT-proBNP; plus CBC (WCC, Hb), metabolic, lipid profile, glucose, HbA1c, insulin.	Insulin: immunoenzymometric assay (IMMULITE 2000); Roche latex assay; Randox automated immunoturbidimetric assay; ECLIA; Innovance vWF activity assay (particle-enhanced turbidometric)
Cruz T et al. (2025) (21)	Spain	Cohort	Adults ≥18 *n* = 113	Non-long COVID	Persistence (>2 months) of symptoms 3 months after onset, not explained by alternative diagnosis; LC group is defined as non-pulmonary symptoms with DLCO >80% predicted at 12 months	SF-36	Serum; Plasma	Complement (C3, C4, CH50), immunoglobulins (IgG, IgA, IgM), autoantibodies (ANA, anti-cytoplasmic patterns; antigen-specific autoAbs; anti-IFN), inflammatory, organ damage proteins, GDF-15 and WFDC2	Turbidimetric immunoassays; indirect immunofluorescence; PMAT (Aptiva); Luminex; Olink multiplex proximity extension assays; ELISA
Fricke et al. (2025) (19)	Germany	Case–control	Adults ≥18 *n* = 88	Convalescent healthcare workers, previously infected	WHO definition of Long COVID	SF-12; bell disability score	Plasma	PBMC scRNA-seq; monocyte DEGs - IL1B, CXCL2; cytokines, chemokines; anti-SARS-CoV-2 nucleocapsid IgG	scRNA-seq (BD Rhapsody Single-Cell Analysis System, Illumina sequencing); Luminex custom cytokine discovery assay; ELISA
Han et al. (2025) (20)	Austria	Case–control	Adults ≥18 *n* = 100	Recovered COVID without LC symptoms, uninfected, pan-negative for anti-spike	PCR-confirmed prior infection; persistent symptoms leading to LC clinic diagnosis	EQ-5D-3L; EQ-VAS	Serum	Autoantibodies to mAChR3, ETAR, β2-adrenergic receptor, AT1R; plus, Ang (1-7) levels; routine labs also recorded (hs-CRP, IL-6, NT-proBNP, immunoglobulins)	ELISA kits; Roche Elecsys (ECLIA)
Ai et al. (2025) (24)	China	Cohort	Adults ≥18 *n* = 255	Non-long COVID	Adults with prior confirmed SARS-CoV-2 infection (PCR, antigen positive); Long COVID classified by symptom-based subgrouping (MULTI, NEU, CACRB, CAPM) using predefined criteria and questionnaires	EQ-5D-5L; EQ-VAS	Blood; Plasma; Serum	Protein biomarkers: ABHD17A, CSNK1D, PSME4, SYVN1. Subgroup proteins: CRH (MULTI), FPGT (NEU), CBX6 (CACRB), DGKH (MSK, SYST), RBBP4 (CAPM). Metabolic pathway changes (glycerophospholipid, pyruvate metabolism).	Transcriptomics; Proteomics; Metabolomics; Phosphoproteomics; scRNA-seq

ANA, antinuclear antibodies; CBC, complete blood count; CRP, C-reactive protein; DLCO, diffusing capacity of the lung for carbon monoxide; DSQ, DePaul Symptom Questionnaire; ELISA, enzyme-linked immunosorbent assay; ENA, extractable nuclear antigen; EQ-5D-3L, EuroQol 5-Dimension 3-Level; EQ-5D-5L, EuroQol 5-Dimension 5-Level; EQ-VAS, EuroQol visual analogue scale; ETAR, endothelin receptor type A receptor; GAD-7, Generalized Anxiety Disorder-7; HADS, Hospital Anxiety and Depression Scale; hsCRP, high-sensitivity C-reactive protein; ICAM-1, intercellular adhesion molecule 1; IFN, interferon; Ig, immunoglobulin; IL, interleukin; I-FABP, intestinal fatty acid-binding protein; LBP, lipopolysaccharide-binding protein; LC, long COVID; mMRC, modified Medical Research Council dyspnea scale; mtDNA, mitochondrial DNA; NLR, neutrophil-to-lymphocyte ratio; NT-proBNP, N-terminal pro-B-type natriuretic peptide; PBMC, peripheral blood mononuclear cell; PCR, polymerase chain reaction; PROMIS-29, Patient-Reported Outcomes Measurement Information System-29; qPCR, quantitative polymerase chain reaction; scRNA-seq, single-cell RNA sequencing; SF-12, 12-Item Short Form Survey; SF-36, 36-Item Short Form Survey; VCAM-1, vascular cell adhesion molecule 1; vWF, von Willebrand factor; WHO-QoL-BREF, World Health Organization Quality of Life-BREF.

In most studies, definitions of Long COVID align with the criteria of the WHO or Delphi group (16, 25, 23, 19). These definitions require symptoms to persist for at least 12 weeks. Two other studies use cluster-based methods for phenotype (22, 24). One study defines Long COVID according to NICE criteria (18) while another uses study-specific criteria for symptom duration (17). Long COVID is also defined as a clinical diagnosis in specialized clinics (20), and as persistence of symptoms for 3 months after infection with operationalization at 12 months (21) in another study. Previous SARS-CoV-2 infections are confirmed by laboratory tests, including PCR, antigen testing, and serology (17, 18, 19, 21, 20, 24) or by clinical criteria, depending on the design and time period of the study (23). All studies included a control group consisting of either healthy controls with no history of SARS-CoV-2 infection or negative serology, or controls with previous SARS-CoV-2 infections but no persistent symptoms or Long COVID criteria at the time of follow-up. The control groups were matched for age and sex in four studies (16, 18, 19, 21) and additionally matched for risk factors in the remaining studies (5, 17, 22, 23) (Table 2).

**Table 2:** Characteristics of Long COVID and controls in included studies.

Reference	Long COVID						Control					
	Total (*n*)	Age	Female, %	Follow-up time after infection	Key comorbidities, %	Acute COVID-19 severity	Control (*n*)	Age	Female, %	Follow-up time after infection	Key comorbidities, %	Acute COVID-19 severity
Maes et al., (2022) (16)	86	28.4 ± 6.2	27.9	3–4 months	NR	Severity NR; acute-phase PBT; the lowest SpO2	39	28.3 ± 7.6	38.5	NR	NR	NR
Sangkaew et al., (2024) (25)	141	46.0 (IQR 33.0–56.0)	79.4	12–16 weeks	DM 9.9, CVS 6.4, CRD 5.7, CKD 2.1, ND 2.8	Severity NR; hospitalized (2.8%), oxygen (1.4%), ICU (1.4%)	159	40.0 (IQR 31.0–57.0)	54.7	12–16 weeks	DM 3.8, CVS 2.5, CRD 1.3, CKD 1.3, ND 0.6	NoPACS
Durieux et al. (2025) (17)	89	42.92 (IQR 32.12–54.1)	53.93	NR	DM 7.87, HT 20.22, HIV status (+) 21.35	NR	89	40.66 (IQR 28.82–51.49)	53.93	NR	DM 1.12, HT 14.61, HIV status (+) 22.47	NR
Polli et al. (2025) (22)	127	53.6 ± 14.5	44.1	10 months	NR	Mixed severity; hospitalized (91.6%), ICU (36.3%)	231	58.5 ± 12.5	31.6	10 months	NR	NR
Chiu et al., (2025) (23)	85	48 ± 13	75	11 months	CVS 24, Gastrointestinal 8, Renal 2, Depression or anxiety 16	Mixed severity; hospitalized (15.3%), ICU (3.5%), ventilated (2.4%)	26	45 ± 19	62	10 months	CVS 19, Gastrointestinal 8, Renal: 0, Depression or anxiety: 0	NoPACS
Xuereb et al., (2025) (18)	174	45.5 (IQR 35.0–58.75)	60.3	5 months	HT 22, HL 17, DM 10, CRD 16, Obesity 39, CKD 0.6	Severity NR; hospitalized (9.2%)	75	44 (IQR 37.5–56.5)	54.7	NR	HT 17, HL 12, DM 7, CRD 11, Obesity 37, CKD 0	NR
Cruz et al., (2025) (21)	31	51.7 ± 13.6	51.6	12 months	CVS 12.9, Autoimmune 37.5, CTD 0, Obesity 33.3	Mixed severity; hospitalized (100%), ICU: PS (49.0%)	31	57.4 ± 13.3	64.5	12 months	CVS 22.6, Autoimmune 12.5, CTD 4.17, Obesity 20.8	Mixed severity; hospitalized (100%), ICU (41.9%)
Fricke et al., (2025) (19)	44	54 (IQR 19–79)	79.5	NR	HT 36.4, Obesity 15.9, BA 15.9, Depression 15.9, Osteoarthritis 13.6, Migraine 13.6, DM 9.1	Mild–moderate severity, non-hospitalized	44	51 (IQR 26–63)	79.5	NR	NR	NR
Han et al., (2025) (20)	100	41.7 ± 12.7	74	6 months	HT 30.0, HL 18.0,	Mild–moderate severity, non-hospitalized	20	44.8 ± 12.0	70	NR	NR	NR
Ai et al. (2025) (24)	201	NR	42.3	6 months	NR	Asymptomatic, mild severity	54	NR	41.1	6 months	NR	NoPACS

DM, Diabetes mellitus; CVS, Cardiovascular disease(s); CKD, Chronic kidney disease; CTD, Connective tissue disease; CRD, Chronic respiratory disease; HT, hypertension; HL, Hyperlipidemia; BA, Bronchial asthma; ND, neurological disease; HIV, human immunodeficiency virus; NR, not reported; ICU, intensive care unit; NoPACS, no post-acute COVID-19 sequelae; PBT, peak body temperature; PS, pulmonary sequelae.

The most frequently used instruments for assessing health-related quality of life are the SF-36 and SF-12 questionnaires. In three studies, the EQ-5D-5L was used to evaluate the dimensions of mobility, self-care, usual activities, pain, discomfort, anxiety, and depression (20, 24, 25). Durieux et al. (17) used the PROMIS patient-reported outcomes system, which includes key domains such as fatigue, physical function, sleep, pain, anxiety, depression, and social roles. One study used the St. George’s Respiratory Questionnaire to assess respiratory quality of life (23). The main characteristics of health-related quality of life assessment are presented in Table 3.

**Table 3:** HRQoL assessment in included studies.

Reference	QoL instrument	What was reported	Timepoint(s)	Cases and controls	Domains most affected	Quantitative HRQoL findings
Maes et al. (2022) (16)	WHO-QoL-BREF, HAMD, HAMA, BDI-II	WHOQOL-BREF domain scores; HRQoL clustering (normal, moderately low, very low)	3–4 months post-acute phase	Lower HRQoL in Long COVID	NR (domains assessed: physical, psychological, social, environment)	WHO-QoL physical: (27.46 and 16.83); psychological: (25.70 and 16.43); environmental: (33.60 and 22.89); (all *p* < 0.001)
Sangkaew et al. (2024) (25)	EQ-5D-5L; Fatigue Score, Mini-Cog, GAD-7, PHQ	QoL impairment threshold (<80% usual health) used within PCS definition	12–16 weeks post-infection	NR	NR	QoL impairment criterion: 13% (95% CI 9–17)
Durieux et al. (2025) (17)	PROMIS-29; Global Health items	PROMIS domains collected (physical function, fatigue, pain, sleep, anxiety, depression, social roles)	Post-acute (>3 months by case definition)	NR	NR	NR
Polli et al. (2025) (22)	SF-36, 6-min walk test, HADS; DSQ symptom clusters	SF-36 domain scores (8 domains) by group	4–24 months post-infection	Lower SF-36 across all reported domains in Long COVID	Role physical; General health; Vitality; Social functioning	SF-36 general health: (61.55 and 35.93); vitality: (66.09 and 33.37); pain: (76.05 and 42.67)
Chiu et al. (2025) (23)	St George’s Respiratory Questionnaire, COPD Assessment Test, Fatigue Assessment Scale, mMRC dyspnea, Leicester Cough Questionnaire, Post-COVID-19 Functional Status.	SGRQ domain medians (Symptoms, Activity, Impact) and cluster-based profiles; CRP correlated with worse SGRQ	10–11 months	Worse respiratory HRQoL in Long COVID, especially severe cluster; NoPACS milder	Activity and Impact (severe cluster highest); Symptoms also elevated	SGRQ total: (36 and 4), (*p* < 0.001); CAT: (16 and 1), (*p* < 0.001); FAS: (34 and 13), (*p* < 0.001)
Xuereb et al. (2025) (18)	SF-36	Between-group comparisons for SF-36 domains	6 months post-infection	Mostly no significant differences; worse General Health and Role Physical in cases	General health; Role physical	General health: (*p* = 0.027); role physical: (*p* = 0.008); overall self-rated health: (*p* = 0.03)
Cruz T et al. (2025) (21)	SF-36	SF-36 administered	12 months	NR	NR	NR
Fricke et al. (2025) (19)	SF-12; Bell Disability Score	SF-12 and disability collected	5–30 months	NR	NR	SF-12 MCS: *t* (20)= − 13.17, (*p* < 0.001); PCS: *t* (20)= − 15.33, (*p* < 0.001)
Han et al. (2025) (20)	EQ-5D-3L, VAS	EQ-5D domain distributions reported within LC subgroups	7–8 months post-infection	NR	Usual activities, Pain, discomfort	Usual activities: (63%/11 and 78%/7.3%) (some problems/unable; orthostatic intolerance vs. no orthostatic intolerance), (*p* = 0.468); pain, discomfort: (95 and 90%), (*p* = 0.143)
Ai et al. (2025) (24)	EQ-5D-5L	EQ-5D-5L, EQ-VAS collected	6 months	NR	NR	EQ-VAS: (64.8 and 89.7) (MULTI and NLC), (*p* < 0.001); (68.6 and 89.7) (CACRB and NLC), (*p* < 0.001)

In most studies, biomarkers were assessed in serum or plasma samples (16, 17, 22, 23, 21, 19, 20, 24), whereas two studies analyzed whole blood samples (25, 18). The duration of follow-up and the timing of sample collection after acute infection varied significantly across studies, ranging from 3–4 months (25) to 6–10 months (18) and up to 12–24 months. ELISA was used in five studies (16), while routine clinical laboratory tests were also performed in some studies (22). Chiu et al. (23) also performed serum autoantibody screening using the IMTEC-ANA-LIAMAX assay to detect signs of autoimmune activity in Long COVID patients.

The characteristics of patients and control groups in 10 studies are presented in Table 2. The total number of participants in the patient group was 1,078, and the control group had 768 participants. All studies included adults aged 18 years or older, with a mean or median age ranging from 28.3 to 58.5 years. The proportion of males ranged from 20.5 to 72.1% across all studies, with at least 20% of participants being male. Comorbidities were reported inconsistently across studies, but metabolic, cardiovascular, respiratory, psychiatric, and autoimmune conditions were commonly reported in patients. In the control groups, the most commonly reported comorbidities were also diabetes mellitus (17, 18, 25), arterial hypertension (17, 21), obesity (18, 21), chronic respiratory diseases (18, 25), and chronic kidney disease (18, 23).

The severity of the acute phase was assessed differently in all included studies. Some groups consisted primarily of out-of-hospital patients with mild to moderate acute infections, while others included patients with mixed severity or who had previously been hospitalized. Several studies reported hospitalization, admission to the intensive care unit, or oxygen demand as an indirect indicator of severity only. This heterogeneity was taken into account when identifying violations in the assessment of quality of life and their relationship to biomarkers in different studies.

### 3.4 Health-related quality of life findings and their associations with biomarker profiles

All the studies used heterogeneous, validated tools to assess HRQoL, including the SF-36, SF-12, WHOQOL-BREF, EQ-5D-3L, EQ-5D-5L, and EQ-VAS. In one study, PROMIS-29 data reported by patients regarding disease symptoms were used (17). Despite methodological heterogeneity, most studies reported a significant decrease in HRQoL among patients with Long COVID. The domains most often affected were physical functioning, vitality, fatigue, limitations in role or routine activities, pain, discomfort, and respiratory distress. Overall, these findings suggest that the decrease in HRQOL during Long COVID is primarily reflected in declines in physical and functional ability, rather than in consistent impairment across all domains. A few studies have reported on the direct association between biomarker profiles and HRQoL.

The most comprehensive analysis was conducted by Maes et al. (16), showing that lower WHOQOL-BREF scores are associated with higher neurotoxicity scores, lower calcium levels, and acute phase severity markers, including increased body temperature peaks and lower oxygen saturation. The WHOQOL-BREF physical score decreased (from 27.46 to 16.83), the psychological score (from 25.70 to 16), and the environmental score (from 33.60 to 22), across normal, moderately low, and very low quality of life subgroups (*p* < 0.001) (Table 3). In the study by Han et al. (20), patients with Long COVID had impairments in usual activities (11%), and pain or discomfort were reported by almost all respondents who filled in the available questionnaires (95%). A positive but not statistically significant correlation was observed between ETAR autoantibody concentrations and impairment in usual activities in the EQ-5D-5L (*r* = 0.33, *p* = 0.099).

In the study by Xuereb et al. (18), HRQoL was assessed using the SF-36, and patients with Long COVID scored significantly worse than controls on several domains of HRQoL, including vitality, physical function, bodily pain, emotional function, social function, and mental health. Worse scores were also seen in terms of overall health (*p* = 0.027) and physical health (*p* = 0.008). Although higher levels of CRP were associated with worse HRQoL in the medium term, no formal modeling of biomarker levels for specific domains of HRQOL was performed.

In the study by Chiu et al. (23), the HRQoL was assessed using the SGRQ. This included the total score, as well as the domains of symptoms, activity, and impact, as well as additional functional parameters such as CAT, FAS, mMRC, LCQ, and PCFS. Cluster analysis based on the SGRQ identified a subgroup with substantially worse CAT scores (16 compared to 1, *p* < 0.001) and higher fatigue scores on the FAS (34 compared to 13, *p* < 0.05). Worsening SGRQ scores were associated with signs of systemic inflammation, such as elevated CRP levels, more frequent presence of anti-SS-B/La antibodies, elevated endothelial markers such as ICAM-1 and VCAM-1, and longitudinal assessment demonstrated a clinically significant improvement in 43% of patients according to the MCID criteria. This improvement was associated with decreases in IL-8 and anti-SS-B/La levels. Studies that used a phenotype-based stratification, including Ai et al. (24) and Polli et al. (22) studies, also found similar results.

In Ai et al. (24), the self-reported health on the EQ-VAS was lower in the Long COVID subgroups than in the controls, particularly in the multi-systemic subgroup, with a score of 64.8 compared to 89.7 in the controls, and in the cardiocerebral subgroup with a score of 68.6 compared to 90.1 (*p* < 0.001). Polli et al. (22) reported consistently lower SF-36 values in symptomatic Long COVID clusters than in asymptomatic controls, including general health (35.93 compared with 61.55), vitality (33.37 compared with 66.09), and pain (42.67 compared with 76.05).

In the study by Sangkaew et al. (25), the association between biomarkers and HRQoL was more interpretable at the level of specific clinical phenotypes, rather than at the population level of patients with Long COVID. In particular, data suggest that impairment of HRQoL during Long COVID is not homogeneous biologically, but consists of heterogeneous phenotypes characterized by different inflammatory, autoimmune, endothelial, or metabolic biomarker profiles.

### 3.5 Biomarker domains reported across included studies

The studies reviewed investigated biomarkers in multiple primary biological processes, including immune, inflammatory, endothelial, coagulation, autoimmune, complement, metabolic, cardiometabolic, intestinal barrier functions, and omics-based molecular markers (Supplementary Table 4). Immune and inflammatory markers were studied in all studies, and the most common were CRP, hsCRP, IL-6, TNF-*α*, IL-1β, and IL-10. Several studies also measured endothelial dysfunction and coagulation markers, such as D-dimer, ICAM-1, and VCAM-1. As reported by Chiu et al. (23), Long COVID patients’ serum levels of ICAM-1 and VCAM-1 were significantly higher than in controls (*р* < 0.05). Less commonly studied were autoimmune and complement markers, which included ANA, ENA, anti-SSB/La (43 to 13%), complement components (C3, C4, and CH50), antigen-specific autoantibodies, IFN antibodies, and GPCR antibodies.

According to Cruz et al. (21), an extended immunological analysis revealed differences in immune profiles between Long COVID patients and control groups including higher IgM levels (1.08 compared with 0.96 g/L, *p* = 0.032), a higher IgG, IgA ratio (6.51 compared with 4.68 g/L, *p* = 0.013), and a higher IgM, IgA ratio (0.63 compared with 0.48 g/L, *p* = 0.012); anti-nuclear autoreactivity also tended to be more frequent in Long COVID (22.6% compared with 3.2%, *p* = 0.058). Metabolic and cardiometabolic markers, such as glucose, insulin, HbA1c, troponin, NT-proBNP, and ox-LDL, were also studied in several studies. In a study conducted by Polli et al. (22), the PEM-fatigue cluster showed higher troponin T levels than in the asymptomatic control group (14.19 compared with 11.26 ng/mL, *p* = 0.029), indicating a phenotypically specific cardiometabolic signal.

**Figure 2:**
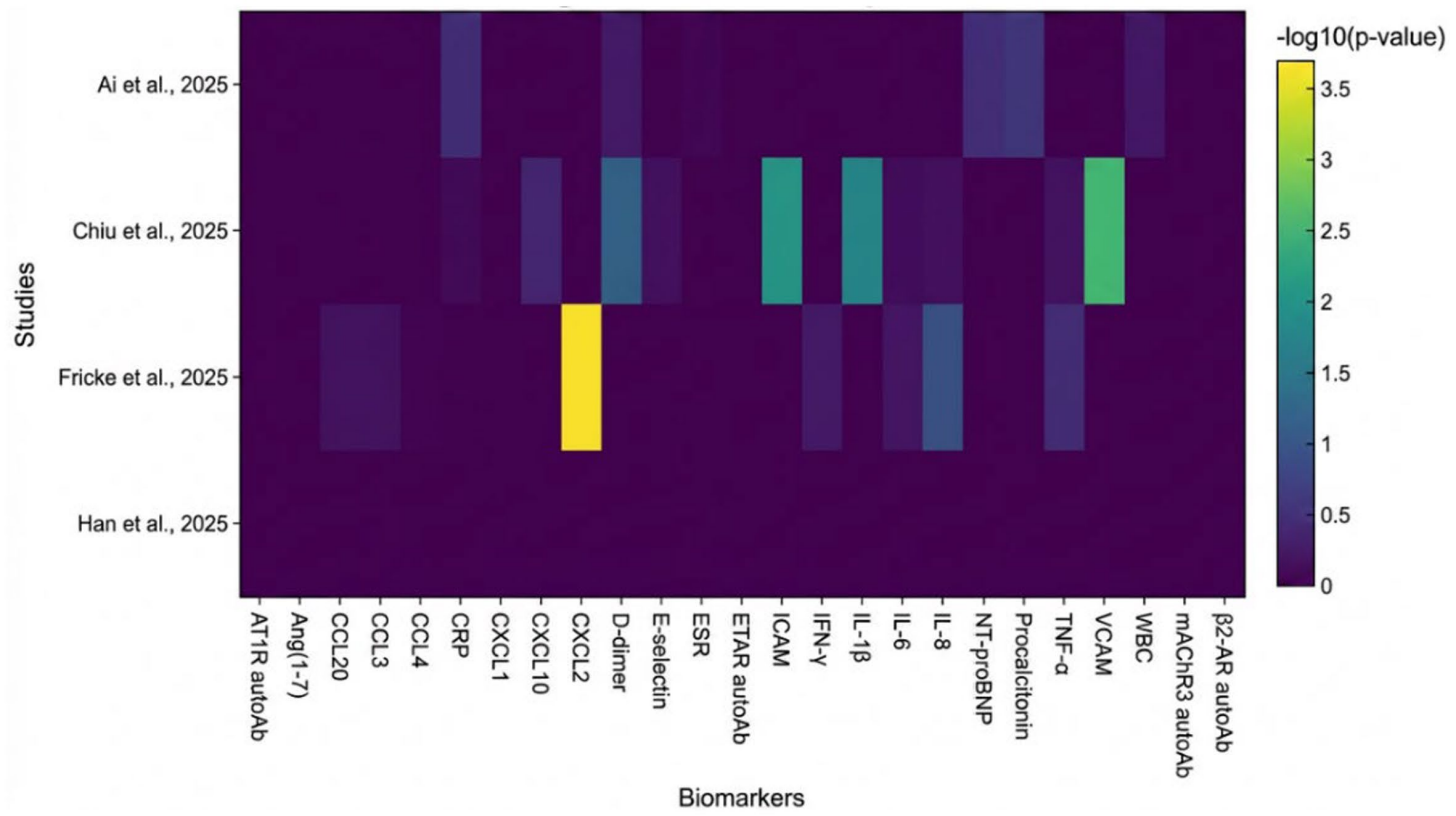
Heatmap of statistical significance (-log10 p-values) for biomarker differences across selected studies.

Durieux et al. (17) investigated zonulin, I-FABP, LBP, and *β*-D-glucan and found that higher levels of zonulin (OR = 1.91, 95% CI 1.23–2.98) and β-d-glucans (OR = 1.80, 95% CI = 1.22–2.7) were associated with Long COVID, while higher levels of I-FABPs were associated with poorer arterial elasticity. Multidimensional molecular approaches, including proteomics, transcriptomics, methylation markers, telomere length, and copies of mitochondrial DNA, were used in four studies. These results indicate that Long COVID may encompass a broader biological heterogeneity, which becomes more apparent after phenotypic stratification (Figure 2).

Overall, the map showed pronounced heterogeneity in biomarker signals between studies. Most notable differences were often seen for markers of systemic inflammation and endothelial dysfunction, while autoimmune, metabolic, and genomic signals were more varied and specific to each study. This suggests that there is no single universal biomarker profile for Long COVID, supporting the idea of the biological heterogeneity of the condition.

## 4 Discussion

This systematic review included 10 studies that simultaneously assessed biomarkers and health-related quality of life in patients with Long COVID. The included studies consistently reported reduced health-related quality-of-life indicators such as physical functioning, energy, fatigue, pain, or discomfort, as well as the ability to perform daily activities. Although the degree and nature of impairments varied from study to study, the most significant impairments were seen in these areas. At the same time, reviewed studies described abnormalities in biomarkers related to inflammation, endothelial function, blood clotting, immunity, and intestinal barrier function. Quantitative analysis of the relationship between biomarkers and quality-of-life indicators has only been presented in a limited number of studies. In most cases, the quality-of-life indicators and biomarker values are studied simultaneously, or patients with Long COVID are compared to control groups without establishing a relationship between biomarkers and HRQoL indicators. The totality of available data suggests a possible biological contribution to the deterioration in quality-of-life, but it does not yet allow us to determine which biomarkers are associated with this deterioration.

The studies included in this review used various tools to evaluate HRQoL, such as EQ-5D-3L, EQ-5D-5L, SF-36, SF-12, WHOQOL-BREF, SGRQ, and PROMIS. Despite differences in assessment approaches, the results were generally consistent, indicating mainly a deterioration in physical and functional aspects of quality-of-life. However, comparability between studies remained limited due to the fact that in some studies HRQoL was measured as a dichotomous outcome rather than continuously. This emphasizes the need for standardization of tools and methods of presenting results for future research.

**Figure 3:**
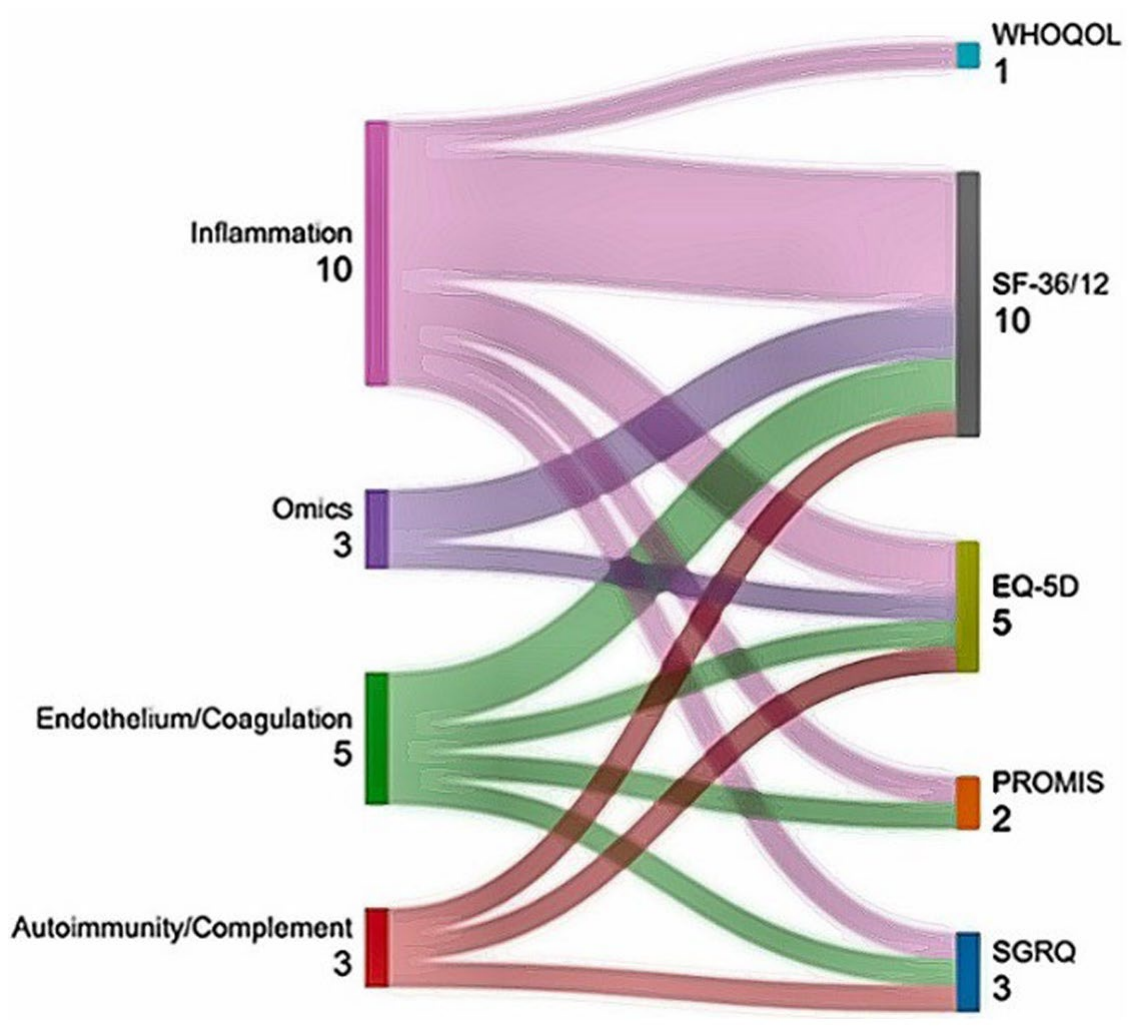
Sankey diagram of the distribution of included studies across quality-of-life assessment instruments and biomarker domains.

Among the biomarkers presented in the included studies, the most frequently assessed biomarkers were markers of systemic inflammation, namely CRP, hsCRP and IL-6. These are followed by endothelial and coagulation markers such as D-dimer, ICAM-1, VCAM-1 and vWF. Signals associated with autoimmune processes, complement activation and broader omics profiles are much less common in the included studies. This distribution is also reflected in the evidence base map (Figure 3), where inflammatory and vascular endothelial domains were most often assessed, while other biological domains are represented in fewer studies.

Several studies included in the review noted that patients with more pronounced symptoms had less favorable biomarker scores, especially related to inflammation, vascular and endothelial conditions. However, the relationship between HRQoL and biomarkers was not assessed directly in most cases. Instead, clinical subgroups, clusters of symptoms, or phenotypes were compared. Therefore, current data indicate that an imbalance of biomarkers may be associated with more severe clinical and functional disorders, leading to a decrease in quality-of-life.

Other studies included in this review on autoimmune and complement-associated markers also show this phenotypic relationship. Although there were significantly fewer studies of this type than those on inflammatory and vascular, endothelial domains, the few studies that were included showed that autoantibody levels differed between clinical subgroups of Long COVID. In some cases, these levels were combined with less favorable clinical profiles or worse HRQoL scores. Therefore, in the case of respiratory phenotypes of Long COVID, ENA reactivity, including anti-SS-B/La, is more often detected in patients with less favorable courses and decreases as the condition improves. Similarly, a higher ETAR level is associated with more restrictions in daily activities on the EQ-5D-3L scale in one study. Taken together, these data suggest that autoimmune signals are more important for individualizing phenotypes of Long COVID than for directly linking them to HRQoL.

Phenotypic heterogeneity was especially noticeable in studies that used multi-genomic approaches. So, in the study by Ai et al. (24), a comprehensive multiomic design was applied, including transcriptomics, proteomics, metabolomics, phosphoproteomics and single-cell RNA sequencing. Patients with Long COVID were stratified into several symptom-associated phenotypes (MULTI, NEU, CACRB, MSK + SYST and CAPM). HRQoL assessment using EQ-5D-5L and EQ-VAS showed that individual phenotypes are characterized by a more pronounced decrease in self-assessment of health status, while corresponding to various molecular patterns including phenotype-specific protein signals and changes in metabolic pathways, such as glycerophospholipid and pyruvate metabolism. In the study by Polli et al. (22), similar conclusions were shown. No differences were found in most biomarkers when comparing patients with Long COVID with an asymptomatic control group directly. More informative results were obtained after clustering by symptoms. The phenotype of post-exercise malaise and fatigue was associated with cardiometabolic markers, highly sensitive troponin, and markers of cellular aging, such as telomere length and the number of copies of mitochondrial DNA, in the PBMC. Inflammatory markers played a less prominent role in this phenotypically oriented analysis, confirming that HRQoL reduction and functional limitations in Long COVID may be more closely related to specific clinical phenotypes than universal changes in systemic biomarkers.

The idea of the connection between the phenotype and biomarker signals was further confirmed in studies using metabolic and multidimensional approaches, including multi-omics approaches, cellular markers of aging, and single-cell transcriptomics. These data indicate that Long COVID, within the studies included, manifests itself as a clinically and biologically heterogeneous condition, where different phenotypes are accompanied by different molecular profiles. These differences may explain why certain common biomarkers or cytokines do not always have a stable association with HRQoL. When analyzing heterogeneous groups of patients, potentially significant biological signals may be smoothed out, limiting the interpretation of results without first classifying patients according to their phenotypic characteristics.

Several limitations were noted in this review. The first is that many studies lacked direct analytical models that examined the relationship between biomarkers and health-related quality of life. This largely reflects the methodology of the primary studies, in which biomarkers and HRQoL were measured within the same cohort, but analyzed separately. In most cases, the studies relied on group comparisons or stratification based on phenotypes rather than regression or correlation models that directly assessed the associations between biomarkers and HRQOL. The second limitation is inconsistency in reporting the severity of acute phase, which reduces the ability to distinguish between complications after severe acute COVID-19 infection and persistent symptomatic syndromes following initial mild or moderate infections. This difference may be significant because the severity of an acute phase can influence both the subsequent decline in HRQoL and abnormal biomarkers. Thirdly, there was significant heterogeneity in HRQoL measurements, follow-up duration (from 12–16 weeks to 24 months), and laboratory methods. In general, these limitations reduced the comparability of various studies and limited the possibility of quantitative synthesis, supporting the use of a structured narrative approach.

From a public health perspective, the findings highlight the need for closer relationships between patient-centered and biological indicators in future follow-ups of patients with Long COVID. A consistent decrease in HRQoL in included studies indicates significant functional burden from this condition. For more accurate interpretation of biomarkers, standardized assessment tools, phenotypic stratification, and longitudinal studies are needed in which biomarkers and HRQoL are analyzed in a single analytical model.

## 5 Conclusion

In conclusion, studies conducted a comprehensive study of biomarkers and HRQoL in Long COVID consistently showed a decrease in quality of life. At the same time, the results for biomarkers were diverse and most often related to inflammation and vascular and endothelial conditions. The direct relationship between biomarkers and HRQoL has been limited, indicating the need for standardized, phenotypically stratified, and long-term studies that combine patient data and biological data.

## Data Availability

The original contributions presented in the study are included in the article/Supplementary material, further inquiries can be directed to the corresponding author/s.
