# Supplementary material for: Long COVID and health-related quality of life: a systematic review of immune, inflammatory, and metabolic markers
Source: Front Public Health. 2026 Jun 17;14:1846407. doi: 10.3389/fpubh.2026.1846407 (PMC13325420; doi:10.3389/fpubh.2026.1846407)
Supplement: Supplementary file 1 [file Table_1.DOCX]

| **Database** | **Search strategy** | **Limits** |
| --- | --- | --- |
| **PubMed** | ("Long COVID"[Title/Abstract] OR "post-COVID syndrome"[Title/Abstract] OR "post-acute COVID"[Title/Abstract] OR "post-acute sequelae of COVID"[Title/Abstract] OR PASC[Title/Abstract] OR "post COVID condition"[Title/Abstract]) AND (biomarker*[Title/Abstract] OR cytokine*[Title/Abstract] OR inflammation[Title/Abstract] OR "inflammatory marker*"[Title/Abstract] OR metabolite*[Title/Abstract] OR metabolomics[Title/Abstract] OR "metabolic profiling"[Title/Abstract] OR "amino acid"[Title/Abstract] OR tryptophan[Title/Abstract] OR kynurenine[Title/Abstract] OR "KYN/TRY"[Title/Abstract] OR TMAO[Title/Abstract] OR "trimethylamine N-oxide"[Title/Abstract] OR choline[Title/Abstract] OR betaine[Title/Abstract] OR carnitine[Title/Abstract]) AND ("quality of life"[Title/Abstract] OR QoL[Title/Abstract] OR fatigue[Title/Abstract] OR "fatigue syndrome"[Title/Abstract] OR symptoms[Title/Abstract] OR "clinical symptoms"[Title/Abstract] OR "cognitive impairment"[Title/Abstract] OR "mental health"[Title/Abstract]) AND (serum[Title/Abstract] OR plasma[Title/Abstract] OR blood[Title/Abstract]) | Publication date: 2020/01/01 to 2025/12/31 |
| **Scopus** | TITLE-ABS-KEY ("Long COVID" OR "post-COVID syndrome" OR "post-acute COVID" OR "post-acute sequelae of COVID" OR PASC OR "post COVID condition") AND TITLE-ABS-KEY (biomarker* OR cytokine* OR inflammation OR "inflammatory marker*" OR metabolite* OR metabolomics OR "metabolic profiling" OR "amino acid" OR tryptophan OR kynurenine OR "KYN/TRY" OR TMAO OR "trimethylamine N-oxide" OR choline OR betaine OR carnitine) AND TITLE-ABS-KEY ("quality of life" OR QoL OR fatigue OR "fatigue syndrome" OR symptoms OR "clinical symptoms" OR "cognitive impairment") AND TITLE-ABS-KEY (serum OR plasma OR blood) | PUBYEAR> 2019 AND PUBYEAR <2026 |
| **Web of Science** | ("Long COVID" OR "post-COVID syndrome" OR "post-acute COVID" OR "post-acute sequelae of COVID" OR PASC OR "post-COVID condition") AND (biomarker* OR cytokine* OR inflammation OR "inflammatory marker*" OR metabolite* OR metabolomics OR "metabolic profiling" OR "amino acid" OR "tryptophan metabolism" OR kynurenine OR "KYN/TRY" OR TMAO OR "trimethylamine N-oxide" OR choline OR betaine OR carnitine) AND (serum OR plasma OR blood) AND ("LC-MS" OR "LC-MS/MS" OR "mass spectrometry" OR "metabolomic analysis" OR ELISA OR "flow cytometry") | No explicit date filter shown here; should be added if applied consistently |

**Supplementary Table 1. Search Strategies**

Search Date: 2026/01/01

**Supplementary Table 2. PECO framework**

| **Component** | **Inclusion criteria** | **Exclusion criteria** |
| --- | --- | --- |
| **Population** | Adults (≥18 years) with Long COVID, PASC, defined according to established criteria (e.g., WHO, NICE, CDC) or clearly described study-specific definitions consistent with the post-acute disease course. Participants must have persistent, recurrent, or new symptoms≥12 weeks after acute SARS-CoV-2 infection, confirmed by laboratory testing (PCR, antigen, serology) or clinical diagnosis.  Studies must report extractable data on at least one immune, inflammatory, or metabolic parameter and/or HRQoL measured using validated instruments (e.g., SF-36, EQ-5D, PROMIS, or comparable tools). | Children and adolescents (<18 years), pregnant women, studies focused solely on the acute phase of COVID-19 (≤12 weeks after infection), animal or in vitro, ex vivo studies, and studies lacking relevant biomarker or HRQoL data. Studies was also excluded when outcomes primarily reflect pre-existing conditions (e.g., active cancer treatment) without separate analysis of Long COVID, PASC. |
| **Exposure** | No intervention was assessed. Exposures of interest include post-acute immune, inflammatory, and metabolic parameters measured in blood, and outcomes include HRQoL. | Exposures measured exclusively during the acute phase of COVID-19; measurements derived solely from non-blood sources (e.g., urine, saliva, feces, cerebrospinal fluid, tissues), imaging studies, or non-validated laboratory methods unless serum, plasma results are reported separately. |
| **Comparison** | Comparison groups included adults (≥18 years) who have recovered from confirmed or clinically diagnosed COVID-19 with resolution of symptoms ≥12 weeks post-infection, and healthy individuals without a history of COVID-19 confirmed by clinical history and/or laboratory testing. | Children and adolescents (<18 years), individuals with Long COVID, PASC without available comparator data, groups limited to the acute phase of COVID-19, and studies lacking serum, plasma immune, inflammatory, or HRQoL data or using unclear laboratory methods. |
| **Outcomes** | ***Main outcomes.*** Health-related quality of life in adults with Long COVID, PASC, assessed using validated patient-reported instruments (e.g., SF-36, SF-12, EQ-5D, WHOQOL-BREF, PROMIS), measured ≥12 weeks post-infection and at follow-up where available.  ***Additional outcomes.*** Fatigue severity assessed using validated instruments (e.g., FSS, CFQ-11, PROMIS Fatigue), with effect measures reported as MD, SMD or RR, OR.  Key symptoms and functional outcomes, including dyspnea and functional limitation (e.g., mMRC, PCFS), were assessed ≥12 weeks post-infection and at follow-up, with effect measures reported as MD, SMD or RR, OR. | Lack of biomarker data or quality-of-life assessment |

**Supplementary Table 3. Risk of bias**

| **Reference** | **Study design** | **RoB tool** | **Overall RoB** | **Main concern(s)** |
| --- | --- | --- | --- | --- |
| Durieux JC et al., 2025 | Case-control | JBI Case-Control | Low | Residual confounding possible despite PSM |
| Xuereb R-A et al., 2025 | Case-control | JBI Case-Control | Moderate | Confounder adjustment details unclear |
| Fricke F et al., 2025 | Case-control | JBI Case-Control | Moderate | Control heterogeneity; adjustment details unclear |
| Han E et al., 2025 | Case-control | JBI Case-Control | Moderate | Multi-control comparability and adjustment unclear |
| Maes M et al., 2022 | Case-control | JBI Case-Control | Moderate | Retrospective acute-phase data; adjustment unclear |
| Visvabharathy L et al., 2022 | Cohort | JBI Case-Control | Moderate | PRO subset size; adjustment/comparability details unclear |
| Polli A et al., 2025 | Cohort | JBI Cohort | Moderate | Mixed recruitment populations; attrition/adjustment unclear |
| Chiu MN et al., 2025 | Cohort | JBI Cohort | High | Attrition at follow-up is substantial |
| Cruz T et al., 2025 | Cohort | JBI Cohort | Moderate | Adjustment and attrition details unclear |
| Sangkaew S et al., 2024 | Cohort | JBI Cohort | Moderate | Adjustment/attrition details need confirmation |

**Supplementary Table 4. Quantitative biomarker findings in the included studies**

| **Study** | **Biomarker domain** | **Comparator** | **Key quantitative biomarker findings** |
| --- | --- | --- | --- |
| Maes et al. (2022) | Inflammation; neuroimmunotoxicity; acute-phase severity | Long COVID QoL clusters (normal, moderate low and very low WHO-QoL) | PBT: (37.07, 38.30 and 38.75), p<0.001;  lowest SpO2: (94.86, 91.62 and 90.37), p<0.001;  TO2 index: (−0.880, 0.218 and 0.628), p<0.001;  NLRP3: (−0.406, 0.030 and 0.347), p=0.002;  OSTOX: (−0.380, 0.140 and 0.269), p=0.005;  NT: (−0.625, 0.188 and 0.419), p<0.001;  NT+TO2: (−0.857, 0.240 and 0.589), p<0.001 |
| Sangkaew et al. (2024) | Routine laboratory, inflammatory biomarkers | PCS and no PCS; PCS clinical clusters | ALP: adjusted OR 1.02 (95% CI 1.00–1.03), p=0.016,  hematocrit: crude OR 0.91 (95% CI 0.85–0.96), p=0.001;  IgG: crude OR 0.78 (95% CI 0.62–0.96), p=0.024;  CRP, IL-6, D-dimer: not significantly associated with PCS. |
| Durieux et al. (2025) | Inflammation; gut integrity; endothelial dysfunction | Long COVID and COVID-positive without Long COVID;  COVID-positive and COVID-negative | oxLDL ≥54,696.07 U/L was associated with aOR 5.78 (95% CI 3.15–10.62), p=0.0001;  zonulin ≥38,426.04 ng/mL with aOR 1.91 (95% CI 1.23–2.98), p=0.004;  BDG ≥372.2 pg/mL with aOR 1.95 (95% CI 1.22–3.11), p=0.005;  VCAM: aOR 0.36 (95% CI 0.15–0.88), p=0.02 |
| Polli et al. (2025) | Cellular aging, cardiometabolic markers; inflammation | Long COVID PEM-fatigue cluster and asymptomatic controls | Troponin T HS: (14.19 and 11.26 ng/mL), corrected mean difference 6.933 (SE 2.61), p=0.029;  telomere length (T/S ratio): (0.79 and 1.07), corrected mean difference −0.272 (SE 0.077), p=0.012;  mtDNA copy number: (0.83 and 1.08), corrected mean difference −0.249 (SE 0.08), p=0.042;  CRP: (6.26 and 5.56 mg/L), p=0.994;  LINE-1 DNA methylation: (74.52% and 75.76%), p=0.441 |
| Chiu et al. (2025) | Inflammation; endothelial dysfunction; autoimmunity | PACS clusters and NoPACS controls; longitudinal improved and unchanged, worsened PACS | CRP: elevated in clusters 3, 4 and NoPACS, p<0.05;  IL-1β, IL-6, IL-8, TNF-α: elevated in clusters 3, 4 and NoPACS, p<0.05;  ICAM-1 and VCAM-1: elevated in all PACS clusters and NoPACS, p<0.05;  anti-SS-B/La positivity: 19% in NoPACS and 42% in cluster 3 and 50% in cluster 4, p<0.05 |
| Xuereb et al. (2025) | Inflammation; endothelial, coagulation; cardiac injury, stress | COVID-positive cases and COVID-negative controls | hsCRP: 0.02 (0–0.29) and 0.01 (0–0.08), p=0.03;  vWF: 98 (74.8–149.7) and 98.7 (74.23–131.65), p=0.63;  Troponin I: 4 (3–6) and 4 (3–6), p=0.15;  NT-proBNP: 30 (14.85–75) and 31 (17–73), p=0.61 |
| Cruz et al. (2025) | Persistent inflammation, autoimmunity, organ-damage markers | PS, LC and recovered (Rec) | Total anti-nuclear autoreactivities: 23.5% in PS and 3.2% in Rec, p=0.033;  22.6% in LC and 3.2% in Rec, p=0.058.  LC immunoglobulin profile:  IgM 1.08 and 0.96 g/L, p=0.032;  IgG/IgA ratio 6.51 and 4.68, p=0.013;  IgM/IgA ratio 0.63 and 0.48, p=0.012.  In PS, inflammatory markers correlated negatively with DLCO: CCL19 R=-0.31, p=4e-05; CCL20 R=-0.26, p=0.00032; CCL3 R=-0.33, p=2e-05; IFN-γ R=-0.29, p=2e-04; IL-8 R=-0.26, p=0.001; MCP-4 R=-0.17, p=0.035.  At 6 months, CCL20 and IFN-γ were already higher in PS (p=0.010 and p=0.026), while from 6 to 12 months CCL19 and CCL3 increased further (p=0.019 and p=0.0004). GDF15 was higher in PS than Rec (p=0.0035) and correlated with DLCO (R=-0.34, p=0.0041); WFDC2 correlated with DLCO (R=-0.28, p=0.015) |
| Fricke et al. (2025) | Monocyte transcriptomics; inflammatory chemokines, cytokines | Long COVID patients and matched convalescent controls | 40 DEGs (37 downregulated), including IL1B, CXCL2, CXCL3, CXCL8, CCL3, CCL4, and TNFAIP3; plasma CXCL2 was reduced (p=0.0002), while CCL20 showed a non-significant downward trend; IL-6, TNF-α, IFN-γ, IL-1β, CXCL1, CXCL8, and CCL4 were below detection thresholds. |
| Han et al. (2025) | Autoimmunity, vascular markers; inflammation | Long COVID with orthostatic intolerance and Long COVID without orthostatic intolerance | ETAR: 16.4 [12.3–22.6] and 16.8 [12.3–44.0], p=0.174;  Beta-2 AR: 12.5 [7.6–16.6] and 14.4 [10.4–29.2], p=0.199;  Ang1-7: 801.6 [547.0–915.2] and 790.6 [588.6–994.5], p=0.339;  ATR1: 113.0 [19.0–927.6] and 144.6 [7.0–1032.0], p=0.955;  mAChR3: 12.8 [9.7–23.5] and 20.1 [11.6–33.1], p=0.310;  hs-CRP: 0.1 [0.0–0.2] and 0.1 [0.1–0.2], p=0.498; IL-6: 1.5 [1.5–2.1] and 1.6 [1.5–2.6], p=0.562;  Leucocytes: 6.5 [5.5–7.6] and 6.2 [5.5–6.9], p=0.031 |
| Ai et al. (2025) | Multi-omics biomarkers; subgroup-specific proteins | Long COVID and non-long COVID (NLC);  Long COVID clinical subgroups and NLC;  12-month relief and non-relief | Long COVID and NLC: ABHD17A 3.12× higher, PSME4 2.11× higher, CSNK1D 20.8× higher, SYVN1 2.59× higher; combined diagnostic model AUC=0.901. Validation cohort: ABHD17A 6.33× higher, PSME4 25.6× higher, CSNK1D 4.13× higher in Long COVID; combined model AUC=0.896.  12-month non-relief: ABHD17A 3.68× higher. Subtype markers/AUCs: CRH for MULTI (AUC=0.92), FPGT for NEU (AUC=0.86), CBX6 for CACRB (AUC=0.91), DGKH for MSK+SYST (AUC=0.93), RBBP4 for CAPM (AUC=0.91) |

In Maes et al., values are presented across the normal, moderate-low, and very-low WHO-QoL groups, respectively.

Unless otherwise specified, values are reported for the study group and comparator group, respectively.

Data are presented as reported in the original studies and may represent means, medians, median [IQR], OR/aOR with 95% CI, corrected mean differences, correlation coefficients (R), fold changes, or AUC.

In Cruz et al., PS refers to pulmonary sequelae, LC to Long COVID, and Rec to recovered controls.

In Han et al., data are expressed as median [IQR].

aOR, adjusted odds ratio; AUC, area under the curve; BDG, β-D-glucan; CI, confidence interval; DLCO, diffusing capacity of the lung for carbon monoxide; hsCRP, high-sensitivity C-reactive protein; IQR, interquartile range; LC, Long COVID; NLC, non-long COVID; OR, odds ratio; PCS, post-COVID syndrome; PS, pulmonary sequelae; QoL, quality of life; Rec, recovered controls; SE, standard error; SpO₂, peripheral oxygen saturation; WHO-QoL, World Health Organization Quality of Life.
